# Extra-Limites

**Published:** 1838-07-01

**Authors:** 


### DISEASES OF THE PULMONARY ORGANS.

Pulmonary affections, during the period under review, were by far the most prevailing diseases. The admissions under this head constituted 11 per cent. of all the cases, medical and surgical, including accidents. The following is the arrangement of the principal of them, in the order of their frequency of occurrence. Phthisis—Bronchitis—Pneumonia—Influenza*—Laryngitis—Pleuritis —Pertussis.

### Phthisis.

*General Statement.*—From a calculation made from the Hospital registers, it appears that, during the years 1836, 1837, 5.29 *per cent.* of the males and 3.70 per cent. of the females admitted were phthisical patients; and that, reckoning both together, 4.43 per cent. of all the cases admitted during that period were of this description. The proportional distribution of the phthisical cases according to *age* was as follows :—

| | | |
|---|---|---|
| 18.85 | per cent. were aged | 20 years or under. |
| 45.08 | . . | from 20 to 30 inclusive. |
| 22.95 | . . | from 30 .. 40 .. |
| 9.01 | . . | from 40 .. 50 .. |
| 3.27 | . . | from 50 .. 60 .. |
| 0.11 | . . | from 60 .. 70 .. |

The deaths from Phthisis (as far as these were recorded in the registers) were 34.66 *per cent.* on the whole number of deaths, or nearly one in three. Among the males 27.65 per cent. and among the females 46.43 per cent. of the deaths were from Phthisis.

The circumstance of the proportional number of Phthisical *admissions* being much greater among the males, is mainly due to a larger number of females than males being admitted for comparatively slight disorders; while, the deaths from *accidents* occurring chiefly among the males, the proportional number of *Phthisical deaths* is thereby lower among the males than the females.

*Analysis of Cases.*—The cases under the immediate care of the author, to which the following observations relate, were sixty-four in number, thirty-seven males and twenty-seven females—comparatively few opportunities occurred of verifying the existence of the disease by dissection; but no cases are included, of the pathological nature of which any reasonable doubt could be entertained.

#### CAUSES.

*Constitutional Predisposition.*—From an enquiry made in thirty-eight instances (twenty-five males and fifteen females) to ascertain whether or not there existed a family predisposition to the disease, there appeared sufficient evidence of such a predisposition having existed in nineteen, exactly one-half, of the cases —of these, eleven were males and eight females; *i. e.* 44 per cent. of the males and 53 per cent. of the females, of whose history any enquiry was made in this particular; if a similar result should ensue on a more extended enquiry, this would show that Phthisis more frequently arises in males independently of constitutional predisposition than in females.

In the nineteen predisposed cases, the disease appeared to be inherited from the parents in thirteen, eight from the paternal, and five from the maternal side

* The Influenza of 1837, prevailed as much at Cambridge as elsewhere, and a large proportion of the hospital patients were affected by it, but comparatively few were admitted patients originally for that disorder.

—in ten, either with or without phthisical inheritance, other members of the family, brothers or sisters, had fallen a sacrifice to the same disease.

The mean age of the predisposed was twenty-two years, and of those in whom no such predisposition seemed to have existed was thirty.

*Intemperance.*—In six (one-sixth) of the male patients hard-drinking might be assigned as a probable cause of the origination of the malady, five of these independently of any family predisposition. In one only of the females could the same cause be assigned.

*Influenza.*—In two of the male cases, the disease originated after an attack of Influenza, in Feb. 1837; in another case (male) the disease became much worse after an attack of the same disorder, and in a fourth (male) the fatal termination ensued on a similar complication.

*Atmospheric Changes.*—Exposure to inclement weather and damp, seemed to have been the exciting causes in two instances (males).

*Child-bearing.*—In four of the female cases the disease either commenced or became confirmed soon after parturition or during protracted lactation.

*Change from a long confinement in bed to a resumption of locomotion.*—In one remarkable instance (female) not a symptom of pulmonary disease declared itself during a long confinement to the horizontal posture for disease of the hip, which terminated by anchylosis; the general health, and moderate embonpoint, except of the affected limb, having been maintained during the whole period; but from the time of the patient's leaving her bed and recommencing locomotion, phthisical symptoms promptly appeared and the disease proved fatal in a few months.

*Violent exertion.*—In two instances (males) the disease made its debut by sudden hæmoptysis occurring after unusual muscular exertion.

*Influence of Age.*—The mean age of the total number (sixty-four) of phthisical patients was twenty-eight; the following table represents their distribution according to age.

| | | | |
|---|---|---|---|
| 20 years or under, | males 8, | females 10, | total 18 |
| from 20 to 40 inclusive, .... | 21, ...... | 17, .... | 38 |
| 40 to 60 ........ .... | 8, ...... | 0, .... | 8 |

Mean age of the thirty-seven males was thirty years and a half, of the twenty-seven females twenty-four years and a half. Mean age of fourteen fatal cases (nine males, five females) was thirty-three; of the nine males thirty-three, and of the five females twenty-one. If these numbers, few as they are, will warrant an inference, it would appear that, in the female, both the attack and the fatal termination occur at an earlier period than in the male.

*Locality of Disease.*—The following statement respecting the *locality* of the disease is introduced in this place, inasmuch as it was conceived to have some bearing upon points connected with its predisposing causes.

Casual observation had led the author to surmise that the disease *commenced* in the right lung * more frequently than in the left, and the object proposed was

* This is not in accordance with statements made by writers of the highest authority, among others by Louis himself, who have come to the conclusion from dissections, that the left lung is more liable to tubercle than the right. The author just named, it seems, was led to such conclusion by the following facts to put this to the test of more accurate enquiry. The results of post-mortem examinations are, upon the whole, less calculated for the determination of this point than the careful investigation of the physical signs of the disease during life, dating from the earliest period of the malady, at which cases may offer themselves to the notice of the enquirer—since by the time the disease has terminated fatally, it has often invaded both lungs to such an extent as to render it impossible to conclude in which it had commenced—whereas, supposing the physical signs, understood as they are at present, to be fair evidence of the presence and situation of the disease, accurate examination by auscultation and percussion during life may suffice, in a large proportion of cases, to determine, if not the exclusive seat, at least the situation of the largest amount of tuberculous deposit.

Where there already existed the *general* symptoms of the disease, dulness on percussion, absolute or comparative in corresponding points of the chest, combined with diminished respiratory murmur or increased vibration of the voice, were accepted as furnishing the lowest but still adequate amount of *direct* evidence of the locality of the disease, in the absence of other of the physical signs.

The following are the results of an enquiry instituted for the above purpose. In 55 cases, 33 male and 22 female, observations were made to determine by auscultation and percussion the principal locality of the disease. For the sake of brevity, the tables exhibiting numerically the distribution of the cases, according to their locality, are omitted, and the results, calculated on the proportions per cent., substituted.

One hundred cases, then, distributed in correspondence with the observations made in the 55 actual cases, would be thus represented, omitting fractions.

In 85, the disease existing exclusively or to the greatest amount in the upper lobe of one or both lungs.

In 15, the disease occupying both lungs equally.

In 56, the disease having commenced, or existing to the greatest amount in the right lung.

In 29, the disease having commenced, or existing to the greatest extent, in the left lung.

But, applying the same analysis to the male and female cases separately, this

---

principally. 1st. That having observed one lung exclusively affected only in seven cases, in five of these it was the left lung that was the solitary seat; and, 2dly, that out of 38 cases in which the upper lobe was entirely occupied by tubercles and excavations, in 28 of these cases it was the left lung that was so affected; but assuredly the first of these facts is numerically too limited to warrant any inference one way or the other; and with respect to the latter, appertaining as it did to the termination of the disease, is it of so much value in determining the point of the priority of, or greater liability to, tuberculous invasion, as observations of the physical signs of the disease, made in its early periods and repeated during its course? Moreover, although priority of invasion of one part of the lungs by tubercles would, cæteris paribus, and more especially if combined with a greater amount of tuberculous deposit, render it probable that that part would be the first excavated—as is in fact the case on comparing the upper lobes with the lower—yet it by no means follows that the *same* external conditions, which might favor the early *deposition* of tuberculous matter in the lungs in one part rather than in another, would accelerate the process of *excavation*, when once softening of the tubercles had overtaken or superseded the process simply of deposition. On the contrary, if the views suggested afterwards in the text be correct, the very opposite result might be anticipated.

disproportion was found to be much greater for the former than for the latter; for in the male cases the disease exhibited this priority of invasion of the right lung over that of the left, in the proportion of 63 to 24, out of 100 cases; in the remaining 13 per cent. no priority being ascertained; whereas, in the female cases, this predominance did not exceed the proportion of 45 to 36, in 100 cases; in 19 per cent. no priority likewise being ascertained.

The following suggestions are hazarded, in explanation of the probable causes thus influencing the locality of the disease, with much diffidence and with every reservation which the limited sphere of the author's investigations requires.

The greater quiescence of one portion of the lungs than of another appears, à priori, a condition likely to favor the earlier deposition in it of tuberculous matter, or, which is the same thing, to dispose to that lesion of its nutrition which terminates in tuberculization. Now, not only does the form of the chest and the mechanism of respiration seem to denote that the upper parts of the lungs have less freedom of expansion in all directions, and undergo in *ordinary* inspiration comparatively less amplification in their dimensions than the inferior lobes—which have the constant play of the diaphragm, as well as the elevation and eversion of the ribs to maintain their expansion—but the mechanical circumstances under which the muscular movements of the superior extremities place the upper portion of the chest, especially in exercises requiring strenuous or sustained efforts, seem to conspire further in rendering the upper lobes of the lungs more quiescent than the lower—for, so far from the muscular contractions and sustained efforts of the superior extremities serving to expand the chest, as is sometimes asserted, they necessarily suppose that the upper part of the chest during these efforts remains more fixed and constant in its dimensions, in order to form a firm support, a *point d'appui*, for the exertion of the required force.—When the same muscles are used as inspiratory instruments, or in extraordinary respiration, the extremities themselves are first fixed, as is seen in persons labouring under orthopnœa, in order that such a function may be possible for them; and, by parity of reasoning, in their ordinary action, they must require that the part of the trunk to which they are attached should be more or less steady and fixed.

If this be accepted in explanation of the well-known fact, that tuberculization commences in the upper lobes, it will serve likewise to account for the additional fact (if it be such) of the priority of the right lung in liability to tuberculization of the upper lobe over the left lung; for, if the muscular action of the superior extremities causes the upper part of the chest to be comparatively fixed during such action, it is obvious that the right side will be more frequently and more steadily fixed than the left, and the upper lobe of the right lung comparatively more quiescent than the upper lobe of the left. The same reasoning perhaps will account for the priority of invasion of the right lung being more frequent among males than females. It has been observed, as stated by Dr. Stokes, that in some persons the respiratory sound is naturally more feeble in the right than in the left lung; this inferiority of sound may be considered as implying, from whatever cause, comparatively less freedom of expansion, habitually, in the act of respiration, which thus may favor the earlier deposition of tuberculous matter.*

---

* Is it possible that the action of the heart, by its alternate expansion and contraction, may add something to the advantage of the left lung over the right, by favoring the continual alternation in the dimensions of its lower lobe especially? The organs situated immediately below the diaphragm seem to contribute to the same result—viz. the greater quiescence of the right lung than of the left: for the permanence of volume and solidity of the right lobe of the liver in the right hypochondrium cannot be so favorable to the expansion of the

In connexion with the present subject a remark suggests itself respecting pleural adhesions—there is no evidence, nor is it generally believed, that these are in all instances, or even most commonly, preceded by an inflammatory condition of the serous membrane; but it is evident that here likewise quiescence of the opposed surfaces of the costal and pulmonary pleura will greatly dispose to diminish the serous secretion, and thus probably favor adhesions—and hence the greater frequency of adhesions of the upper than the lower lobes. A case which lately occurred to the notice of the author, seemed to indicate that the mechanical confinement of the chest might of itself be sufficient to cause the adhesion of the entire periphery of both lungs. A female died suddenly, in whom no disease of the lungs beyond a single *earthy* tubercle in the summit of one of them was found, but there was universal adhesion of the pleura on both sides. From the previous appearance of the patient, and the inspection of the conformation of the chest after death, it was evident that this patient had habitually subjected herself to very tight lacing. That the use of tight stays favors the development of phthisis, must be still more apparent if the above view that is taken of the effect of the quiescence of the lungs be correct.

In conclusion, it is plain if quiescence of the upper part of the chest favors deposition of tuberculous matter in that region, there is on this, as well as other accounts, much room for judicious selection in determining the exercises proper for those predisposed to pulmonary consumption; and that especially such should be avoided as require violent or even sustained efforts in the muscles of the upper extremities, as rowing,* fencing, the use of dumb-bells, &c.—whereas such exercises should be preferred in which the muscular action is general, and distributed without any *stress* being thrown upon the muscles connecting the superior extremities with the thorax; while the necessity of inspiring more frequently and forcibly in consequence of the accelerated circulation, would prove more beneficial than otherwise. For the same reason, the exertion of the voice, as in reading aloud and singing, *previous* to the development of the disease, might assist in preventing it. The common remark of a person having *weak lungs*, however adequately it may denote, in unprofessional language, their condition when already tuberculous, conveys a very erroneous impression, when applied to those perssons in whom only a *general* tuberculous diathesis may be suspected, if by such designation it is insinuated that care should be taken to avoid the full and free exercise of the lungs.

### SYMPTOMS.

*The Hectic.*—Rigors recurring daily with more or less regularity, followed in about one-half of the instances by perspirations, were noted in a third nearly of the cases—they were present in six out of the fourteen cases which remained under observation to their fatal termination; but the mean time the disease had lasted, in those instances in which these phenomena presented themselves as prominent symptoms, was considerably below the mean duration of the disease in the total of the cases at the time of observation, indicating the hectic to be frequent, especially in the earlier stages of the disease.

---

lung placed immediately above it, as the variable capacity and pliability of the stomach in the left hypochondrium. It may be here remarked, that all the circumstances adduced in proof of the greater quiescence of the right lung being a cause of its earlier tuberculization, in a mechanical point of view, might be expected to act reversely in favoring the process of excavation—whence, should it appear that the left lung is more frequently or more early excavated, the difference of the results admit still of being reconciled.

* Rowing has been *recommended* by some, for those liable to phthisis.

In about one-third of the cases in which there were rigors, they recurred daily with periodical precision.

Profuse perspirations occurred in 22 cases, and at least as frequently in those in which the disease was comparatively recent, as in the more advanced stages—they were present in one-half the cases in which there was diarrhœa.

*Emaciation.*—More or less emaciation was observed in 45 of the cases, (about 70 in 100,) but it was excessive only in 27, less than in one-half, and since the mean time that had elapsed since the commencement of the attack in these, on comparison with the general average of the duration of the disease in all, was considerably below the latter, it would appear that this symptom is a measure rather of the actual rate in the progress of the disease, than of its previous duration—it was present in all the fatal cases with the exception of two.

*Hæmoptysis,* had occurred at some period or another in 34 out of the 64 cases (53 in 100), and nearly as frequently in the females as males. Its occurrence was more frequent among those above 30 years of age than those below 30—and this difference was greater among the male than the female cases. In one instance (male) the hæmoptysis was so copious and so frequent as to produce the symptoms usually observed in idiopathic anæmia.

*Expectoration—Quantity.*—The expectoration was noted to be scanty in 26 out of the 64 cases, and in two-thirds of these the duration of the disease had been short of the general average, in one-third it had exceeded it—in 14 only was it noted to have been copious, but in these the disease had lasted beyond the general average : this but agrees with the general statement that the expectoration for the most part becomes more copious as the disease advances. There occurred however some striking singularities in this respect. In one remarkable case, in which there existed every evidence which auscultation and percussion conjointly afford of the presence of a very extensive excavation, with the additional confirmation derived from the general phthisical symptoms, but which did not remain under observation to its termination, the patient while in the hospital never expectorated, and affirmed that he never had during the whole course of the disorder. In another instance the expectoration was scanty throughout, and latterly extremely so, in which after death a considerable anfractuous cavity, with many smaller cavities, and a multitude of crude tubercles were found. In this case, the course of the disease was rapid, not exceeding four months from its invasion to its termination ; there had been more than ordinary suffering from dyspnœa, and the internal organs generally were much congested with blood. May not the absence of expectoration have contributed to hasten the fatal termination, by preventing that balance between the circulating mass and quantity of the excretions, which compensates for the diminished efficacy in the lungs in producing the changes in the blood essential to its due assimilation with the structures of the body, and the maintenance of their growth or volume? In another case, in which the tuberculous matter was infiltrated, and there existed no cavities, but the cervical and bronchial glands were enormously enlarged, and the peritoneum also tuberculated, the expectoration was almost null through the whole course of the disease—and in this case also there was more than ordinary suffering from dyspnœa. In another case, which will hereafter be detailed, where the tubercles in the lungs, with the exception of one or two small cavities, were all crude, but where there was an extraordinary amount of tuberculous matter in other organs, the expectoration had all along been very trifling. In six out of eleven cases, in which pectoriloquy existed, the expectoration was scanty.

*Qualities of Expectoration.*—All descriptions of expectoration were met with, which are ordinarily stated as occurring in phthisical patients, such as frothy, mucous, puriform, &c. In seven cases it was noted as consisting merely of frothy serum, in one of which the disease was found after death to be confined entirely to the miliary form of tubercles ;* but in another instance the same character of the sputa had been observed during life, when after death large excavations were found. With respect to the *form* of the sputa, these were noted as *globular* in 12 instances, and this form appeared to be predominant. Out of 17 cases in which any note was made of the *colour* of the sputa, in 11 it was remarked to be greenish, in three greyish, in two whitish, and in one yellow—the greenish colour persisted in those which remained under observation to their fatal termination.

*Physical Signs.*—By far the most constant of these, as might be expected, were diminution of the respiratory murmur, diminished sonoreity of the chest on percussion, and increased vibration of the voice as conveyed to the ear in auscultation of the chest. As one or other or all of these signs were present in most of the cases, but from accidental reasons frequently no exact mention is made of them in the reports, it is their comparative frequency of occurrence and the frequency of their coincidence, in those cases in which the results of auscultation and percussion are minutely recorded, that merit most notice.

Increased resonance of the voice in some part of the chest, estimated by comparison with the corresponding part of the opposite side, was remarked in 36 cases out of the 64; this includes every degree of increased resonance of the voice, from that which was limited to the communication of greater vibration to the ear or hand applied to the chest, to that producing the most decided pectoriloquy.

Comparative diminution of the respiratory murmur was noted in 29 cases. The condition of the lung which produces this phenomenon had of course in many of the cases been replaced by that more advanced stage which gives rise to other modifications of the respiratory sound, as the various râles, harsh, bronchial, cavernous, sucking respiration, &c.

Diminished sonoreity of the chest on percussion was noted in 30 out of the 64 cases—in all it corresponded to the upper lobes—in 22 to the right, in 11 to the left lung, in three only to both. It was the coincidence of this phenomenon in one or other of the sub-clavicular regions with diminished respiratory murmur and increased vibration of the voice, combined with general phthisical symptoms, which was principally attended to, in detecting the earlier stage of the disease, and from which it was attempted to infer the comparative frequency of priority of invasion in the two lungs respectively. This coincidence, taking dulness on percussion as the term of comparison, was as follows—of the 30 cases in which dulness on percussion was noted, in 14 there co-existed, *in the same situation*, diminished respiratory murmur, and in 21 increased vibration of the voice—also with respect to other auscultatory phenomena, in 12 of the 30 cases the respiration was observed to be in the same situation either harsh, bronchial or cavernous, in 15 there was some modification of râle, and in 3 instances the cough was jarring and successive on applying the ear to the chest.

### COMPLICATIONS OF THE PULMONARY DISEASE.

*Tuberculous Affections of other Organs.*—In one case of an unmarried female, æt. 18, during the whole course of which there was the greatest obscurity in the

---

* The author possesses the notes of another case which occurred several years ago at St. Bartholomew's, in which the patient had had expectoration of the same quality to an enormous extent, and in whom only miliary tubercles of the lungs were found.

physical signs of pulmonary disease, extreme hectic, dyspnœa, cough without expectoration, being the only constant symptoms denoting the existence of phthisis —the granulations of Bayle were found disseminated throughout both lungs, but leaving them everywhere pervious to air; but the greatest amount of thoracic disease occurred in the bronchial glands, which formed an enormous mass compressing both bronchi, but especially the right. Agreeably to the observation of Andral, this condition was indicated by no determinate signs during life: the only sign observed during life which admitted of explanation by the results of necroscopical examination, was the bronchophony which had been frequently but not constantly remarked in the right scapular region, and in the same situation the chest was thought to be less sonorous on percussion than elsewhere. In the same case the cervical and mesenteric glands were similarly and to a great extent diseased, and the omentum converted into a cheesy mass. In another case, which will be given in detail, the very unusual complication (vide comparative table of Louis) of scrofulous disease of the kidney occurred ; in the same case the testes, epididymes especially, and the prostate were in a similar state; the most distinctly tubercular form existed in the testes. Contrary to the assertion of Louis, that the disease is *always* most advanced in the lungs, in this instance the disease was very much further advanced in the kidney. Is it not possible that when, as in this and the last-mentioned case, the tubercles in the lungs do not advance and soften and there has been little or no expectoration, the tuberculous diathesis becomes more pervading and more readily leads to deposition in other organs?

*Pneumonia.*—In two cases there was an intercurrent or terminating attack of pneumonia.

*Syncope.*—In one instance of remarkably acute phthisis, the patient (a female) more than once, some weeks previous to her death, appeared on the point of dying from long-continued syncope—no dissection was made in this case, and there was some suspicion of an attack of pericarditis.

*Ague.*—In one instance the patient suffered in the course of the disease from a smart attack of this disorder.

*Œdema,* occurred in a noticeable degree only in 4 instances.

*Bronchocele,* in a slight degree, formed a complication of the disease in one instance, a female.

*Abdominal Disorders.*—Gastric and intestinal disorders formed a very frequent complication—it was noticed in some form or another in 17 cases. *Diarrhœa* occurred in 12 of the cases ; *peritonitis,* as ascertained by dissection, in two.

AFFECTIONS OF THE NERVOUS SYSTEM.

*Headache* was a very frequent and distressing symptom, especially among the female cases; it occurred as a prominent symptom in 9 instances, 7 of which were of females, *i. e.* about 1 in 4. In one case of phthisis occurring in a child, which is not included in the cases the subject of the present analysis, the disease was complicated with *chorea,* from which she recovered, the phthisical disease advancing in the mean time.

*Delirium* in a slight degree was observed in 4 cases.

*Amenorrhœa* existed in 6 of the cases, making about 1 in 4 of the females of an age to menstruate. In these 6 cases the disease on an average had lasted 7 months.

### COURSE OF THE DISEASE AND RESULT OF THE CASES DURING THE TIME THEY CONTINUED UNDER OBSERVATION.

At the time of the admission of these 64 phthisical patients, the disease on an average, had existed 10 months—26 of these left the hospital alleviated in some degree, more or less: the mean age of these was 26½. In 2 of these 26, males, æt. 16 or 17, the disease which was sufficiently decided both by the physical signs and the occurrence of hæmoptysis, seemed to have been arrested, each having remained a year or more in the enjoyment, apparently at least, of perfect health. In 4 others the patients at the time of their discharge considered themselves as quite well, but still in their aspect and by the persistance of the physical signs betrayed the permanence of the disease.

Twenty-four, whose average age was 29, had become worse at the date of their discharge, or had received no benefit—many of these probably died shortly after their leaving the hospital. Fourteen, whose mean age was also 29, died whilst they remained hospital patients.

The following table shows the distribution of the admissions and discharges, marking the respective results, in the different quarters of the year.

| Spring. | Summer. | Autumn. | Winter. | |
|---|---|---|---|---|
| 20 | 16 | 18 | 10 | Admitted. |
| 6 | 8 | 6 | 6 | Relieved. |
| 8 | 4 | 9 | 3 | Not relieved. |
| 6 | 4 | 3 | 1 | Died. |

From which it would appear, taking into account the proportional admissions, that the Spring and Autumn were the seasons most formidable to the phthisical patients.

### MODE OF TERMINATION OF THE FATAL CASES.

In 6 out of the 14 cases the fatal termination seemed to be due simply to the devastation produced by the original tuberculous disease of the lungs. In one by the *intensity* of the disease, (acute phthisis). In one by the combination of pulmonary congestion. In two by the supervention of pneumonia. In one by excessive diarrhœa and attendant disease of the intestines. In two by the supervention of peritonitis. In one the stress of the symptoms was in the urinary organs, in which the disease was the most advanced.

### MORBID ANATOMY.

*Of the Lungs.*—Under this head the cases afforded little with regard to the proper pulmonary disease that is deserving of further notice than has already been devoted to them in connexion with the history and symptoms. It may be remarked however, that in one instance, where there existed an extensive excavation in the upper lobe, the disease in the remainder of the lungs presented nothing that could be decidedly called tubercular, but exhibited the precise characters of the grey induration described by Laennec as designating chronic pneumonia. Miliary tubercles were the exclusive form of the disease in one case, and in another this form existed alone in one of the lungs, while the other lung contained likewise other larger tubercles, but still in their crude state. In both these instances the lungs were found in a state of pneumonic congestion. In the case already alluded to, remarkable for the amount of disease in the bronchial glands and where the tubercles were all crude, several of the minute bronchial ramifications contained a white matter, which was easily expressed in a vermiform shape and was of a soft caseous consistence—this is obviously favourable to the opinions of Dr. Carswell on the seat of the tuberculous matter of the lungs.

*Of other Organs.*—Tuberculization of the cervical and mesenteric glands, it has already been stated, was found in one of the cases. Peritoneal inflammation evidently of a scrofulous (tuberculous?) nature, was met with twice, *i.e.* in the two cases in which the pulmonary tubercles either presented only the miliary form or were still in a crude state. This complication was not betrayed during life by symptoms of such urgency as attend ordinary peritonitis. The case in which with the pulmonary tubercle there existed a tuberculous state of the kidney, testes, and prostate, will be subjoined in detail. In two cases the mucous coat of the stomach presented the appearance denoted by the French *mammelonnée.*

### TREATMENT.

Although it has been stated that a considerable proportion of the phthisical patients left the hospital more or less relieved, this improvement, it is probable, was in most instances more attributable to the advantages attendant on a change of residence or a proper regimen, than to the palliative effects of the remedies employed.

*Setons.*—If it is fair to conclude that in any of the instances the disease was arrested, when submitted to treatment in its early stage, this desirable event seems to have occurred in an instance in which a seton inserted in the sub-clavicular region formed the principal remedy. In this case, of a youth, who among other phthisical symptoms had had hæmoptysis, the disease has made no further progress up to the present time, an interval of two years.* In two other instances much relief ensued from the same remedy by the abatement of the dyspnœa which it produced.

*Iodine.*—In one case, apparently of incipient phthisis, considerable improvement took place in the patient's general health, while under a course of hydriodate of potash in small doses, but with this remedy was combined a change of air from the town to the country.

With respect to remedies employed as palliatives for particular symptoms, temporary good effects were not unfrequently obtained by them, but they soon became unsatisfactory in their results—and indeed it may be remarked that a *general* hospital is not the best calculated for furnishing even this partial relief to phthisical patients.

*Expectorants.*—The expectoration often *diminished* under the use of various remedies, especially ipecacuanha, and apparently in one instance, creosote; but this result was but too frequently attained at the expense of increasing the dyspnœa and rendering the cough more harassing. It was only therefore where the facility of expectoration was increased without affecting much the quantity, that these remedies appeared to have acted beneficially.

*Emetics.*—Where the hectic or dyspnœa was very urgent, and there was no contra-indicating condition, the exhibition of an emetic, in the few instances in which it was adopted, was attended with more decided though only temporary relief than followed from any other remedies, and this occurred even in cases very far advanced.

*Quinine.*—Sulphate of quinine in an acid mixture was frequently employed to combat the daily return of rigors followed by perspirations, and succeeded so far in about two thirds of the cases in which it was employed. This remedy

---

* Since the above was written, this individual presented himself at the hospital: on examination the dulness on percussion which had been noted under the right clavicle, and the vibration of the voice and diminished respiratory murmur, observed in the same situation, had entirely disappeared, and he had had no pulmonary complaint since his discharge.

seemed to be the only one from which the patients derived any appreciable benefit, under circumstances in which proper regimen and care could not be duly enforced—viz. among the out-patients.

*Cupping,* was sometimes employed with success in arresting hæmoptysis; likewise in one instance the *subacetate of lead,* and in another the *tartarized antimony* seemed to contribute to this result.

Any further analysis of the treatment employed in these cases it is feared, would only confirm the unsatisfactory nature of the results obtained by similar means in the hands of other practitioners.

### APPENDIX.

CASE.—*Crude Tubercles pervading both Lungs—Scrofulous Disease of the left Kidney and Ureter—of the Testes, Epididymes, Vasa Deferentia and Prostate.*

R. T., æt. 24, servant, was admitted in-patient, March 8, 1838. For twelve months he had suffered pain in the back, and during the same period had been subject to some affection of the urinary system, producing frequent micturition, nine or ten times daily; according to his statement he had had no pulmonary complaint till a month previous to his admission, when he became short-breathed, and during the last week only he had been troubled by a cough, which was unaccompanied by expectoration.

At the time of his admission he was much emaciated, and had a hectic aspect. The gastro-intestinal functions were but little disturbed. He principally complained of an aching pain in the lower part of his back and sacrum, and of the whole of the left-thigh, with a weakness of the same limb, on which he could bear no weight, but which he could move when in bed—the other lower extremity had, he stated, been similarly but in a slighter degree affected, and had recovered—he had felt frequent twitchings of the flexors of the left-leg. There was no pain on pressure of the back, except opposite the sacro-iliac symphyses, and here it was slight. A swelling of a cylindrical form, rather firm and not tender, was observed in the course of the right spermatic chord. On a subsequent examination by the surgeon, there was found to be an obstruction to the passage of a bougie into the bladder, which, however, did not appear to be of the nature of a stricture—the urine was generally turbid and of a deep colour, as if containing blood.

He had a short ineffectual cough, was short-breathed, but never had hæmoptysis; neither on the first nor on subsequent examinations was there observed any decided dulness on percussion of the chest, nor any marked resonance of the voice. The respiratory sound was coarse in both subclavicular regions, and posteriorly it was nowhere distinct and natural, but replaced by rhoncus; on an examination a few days previous to his death, a loud creaking was heard under both clavicles, and large crepitation in the inferior lobes of right lung anteriorly—posteriorly large crepitation pervaded both lungs.

No mention need be made of the remedies employed, as they afforded no relief, and he continued without much variation in the symptoms, except increase of the dyspnœa and a rapid advance of the emaciation, till the day of his death, which occurred March 26.

### *Post Mortem Examination Twenty-four Hours after Death.*

*Chest.*—Both lungs were studded throughout with immature tubercles, more numerous in the upper lobes. There were one or two small cavities of the size of hazel-nuts in the centre of the upper lobe of left lung. The left lung was much denser than the right, but this was produced by its being œdematous, especially in the central parts, the circumference remaining crepitous. The right lung was everywhere crepitous. There was sanguineous congestion in the posterior aspect of both lungs.

*Abdomen.*—The swelling in the situation of the right spermatic chord was found to have been produced by a scrofulous abscess between the abdominal muscles which had made its way in that direction. The left kidney was double the natural size, with an externally lobulated form; on its incision, the pelvis and calyces were found very much thickened and distended; the calyx belonging to the lower part of the kidney had, apparently, by its enlargement caused absorption of that part of the organ, and become a large sac, which was distended with a whitish granular fluid; the thickening of the walls of the calyces was produced by a layer of white granular deposit, one-eighth of an inch in thickness. The ureter of the left kidney was very much thickened, enlarged, and filled with cheesy matter, but pervious for urine. There was scrofulous disease of both testicles, which appears to have begun in the epididymis; the disease in the testicle itself was more distinctly tubercular, whereas abscesses existed in the epididymis; both vasa deferentia were much thickened and enlarged, and communicated with a scrofulous abscess of the prostate. The right kidney was slightly granular.

The kidney, testicles, and prostate, are preserved in the anatomical museum, from the catalogue of which the above description is principally taken.

### Bronchitis.

*General Statement.*—The cases of Bronchitis, acute and chronic, constitued 2.69 in 100 of all the admissions during the years 1836, 1837; distributed in the different seasons as follows:—

| | | |
|---|---|---|
| Winter months | 4.34 | in 100 admissions. |
| Spring ..... | 2.75 | ...... |
| Autumn ..... | 2.57 | ...... |
| Summer..... | 1.30 | ...... |

The comparative prevalence, according to seasons, was in the same order for both years.

*Analysis of Cases.*—The following report is drawn up from an analysis of 27 cases under the author's care, including two cases, in which the disease occurred twice in the same individual.

CAUSES.

*Influence of Age.*—Of the 27, 11 were under 20 years of age.
3 between 20 and 40
11 .... 40 and 70
2 (occurring twice.)

*Habit of Body—Antecedent Diseases.*—In three out of ten children, whose ages varied from 16 months to 12 years, there existed a scrofulous habit of body, a chronic defluxion from the nares, among other strumous symptoms, forming an ordinary accompaniment of the bronchial affection.

In two the disease occurred in a cachectic state of the body, consequent on scarlet-fever, with dentition in one instance; and in two (one being also of a scrofulous constitution) it formed the sequela of measles; in one, likewise of a scrofulous habit, it succeeded an attack of influenza. In the adult cases (17) it was seldom that any other predisposing or exciting causes were traceable, beyond those usually assigned for ordinary catarrhal affections—a general cachectic state of the system, combined with thoracic deformity, might be alleged as a predisposing cause in one instance, and an attack of influenza as the exciting cause or debut of another.

*Symptoms.*—In 11 out of the 27 cases the disease presented the acute form, seven of these being of children ; in the remaining 16, it was chronic. As the direct symptoms of the bronchial affection presented in none of the cases anything of unusual occurrence, it is unnecessary to occupy space in the consideration of them; in one instance, however, they were so intense as to threaten immediate suffocation.

*Complications.*—The most frequent complication was *anasarca,* which occurred to a greater or less amount in six cases, including two children that had had scarlet fever : next in frequency of the complications was distressing *palpitation,* met with, independently it seemed of any organic disease of the heart, in three of the patients, all above 30 years of age ; scrofulous *ophthalmia* existed in one instance in a child; some form of *gastro-intestinal disorder,* including one instance of *lumbrici* in a scrofulous subject, occurred in five of the cases ; *convulsions* occurred in one of the children, and *cerebral congestion* formed a dangerous complication in one of the adult cases.

COURSE AND TERMINATION OF THE DISEASE.

*Duration of Treatment.*—The following table represents synoptically the duration of the treatment, in relation to difference of regimen (between out and in-patients), age, and acute or chronic nature of the disease,

| | | | |
|---|---|---|---|
| Mean duration of treatment of | (9) | in-patients discharged recovered, | 24 days. |
| ——— | (12) | out-patients ——— | 34 — |
| ——— | (11) | acute cases (recoveries) ——— | 24 — |
| ——— | (10) | chronic ——— | 37 — |
| ——— | (9) | patients under 12, (recoveries) | 26 — |
| ——— | (12) | ——— above 20 and under 70, | 33 — |

*Results.*—Twenty-one out of the 27 cases were discharged recovered, three benefited, and of the remaining three, the histories were incomplete.

*Mode of Termination.*—The following statements respecting two of the patients, comprises all that the cases offered remarkable under this head. In one case, occurring in a leuco-phlegmatic female, aged 24, during the prevalence of the influenza, the attack, which was acute and severe, and attended with loud bronchial râles, of a sibilous and rhoncous, and also of a mucous character, pervading both lungs, terminated by *resolution,* without there having been at any period the least expectoration. In another case, occurring in a corpulent female aged 40, subject to cerebral congestion, and previously a patient for slight paralysis attended with hysterical affections, the cessation of a severe attack of bronchitis, accompanied with copious expectoration, was promptly followed by symptoms denoting high *cerebral excitement and congestion,* and which required and yielded to active antiphlogistic and counter-irritant remedies.

TREATMENT.*

*Of the Acute Cases.*—In the 7 acute cases, which occurred in infants and children, the principal remedies employed were *emetics,* followed by small doses of ipecacuan, sometimes joined with antimony, where the febrile symptoms were high. In only one instance, of a boy, attacked with very severe bronchitis after measles, *V.S.* from the arm was employed, which had been performed previous

* The author cannot refrain from giving his humble testimony, derived chiefly from the observation and conduct of the cases under review, to the valuable therapeutic instructions in relation to this disease in Dr. Stokes' work on the diseases of the chest.

to his becoming an hospital patient. In another instance, a child aged 5 years, a secondary attack of the different lung appeared to be arrested by the application of a few *leeches*. In only one instance was a *blister* applied in the cases of the children, which was employed for, and removed a partial congestion of the lung which followed the bronchitic attack.

In one of the *adult* acute cases (3), occurring in a haggard old man during the prevalence of the influenza, and a very severe attack, the treatment of which commenced during its advanced stage, the disease yielded to the exclusive use of *tonic remedies*, such as ammoniacum, nitric ether, squills, and latterly quinine. In another case, of a corpulent woman aged 40, with very high fever and tendency to cerebral congestion, the primary stage yielded to a *V. S.* followed by small and repeated doses of tartarized antimony—the advanced stage yielded to the use of ipecacuan, hyoscyamus, and a blister between the shoulders. In the remaining adult case, occurring in a meagre female aged 23, recovery ensued under the antimonial treatment, with the warm bath, followed by ipecacuan as an expectorant.

*Of the Chronic Cases.*—In 5 out of the 10 adult chronic cases, *blisters* were employed and with good effect—they appeared to be of most service when applied between the shoulders. In one very severe case the greatest relief resulted from their repeated application.

*Ipecacuan* was used in several of the chronic cases, but as it was combined with other remedies of more striking efficacy, its share in effecting a recovery cannot be appreciated—in one case however, occurring during the influenza, recovery promptly ensued on its use joined with nitric acid and hyoscyamus.

*Squill*, as a principal remedy, or an adjunct, was used in a large proportion of the chronic cases, and frequently with relief to the symptoms, especially by rendering the expectoration easier.

*Ammoniacum.* By far the most strikingly beneficial results in the treatment of the chronic form of the complaint ensued on the use of ammoniacum mostly combined with squill. In some instances the effects were quite surprizing—in aged persons, who for very many years had in all seasons alike suffered more or less from the disorder, the symptoms yielded to its use, even while they attended as out-patients during the severe weather. In the most memorable instance of recovery that occurred, ammoniacum was at first the only remedy, besides hyoscyamus and small doses of ipecacuan, used, and was quickly followed by manifest and progressive improvement.

*Quinine*, combined with ammoniacum and squills, where the disease attacked debilitated subjects in the chronic form was employed occasionally with apparent benefit.

*Preparations of Iron*, used in one of the cases, occurring in an old man, seemed to assist in restoring the strength.

*Conium and Hyoscyamus* were frequently employed as adjuncts to the expectorants, and were occasionally found equal to the removal of an irritative cough, remaining after the expectoration had ceased or abated.

*Nitric Acid* was sometimes used with apparently good effect, and in one instance of recovery, was, with ipecacuan, the principal remedy.

*Nitric Ether* in very large doses, combined with the warm bath, was in one instance of extreme suffocative orthopnœa attended with prompt relief to the urgency of the symptoms.

### Pneumonia.

*General Statement.*—The cases of pneumonia did not constitute one in 100 of the admissions—more than one-half occurred during the Winter months.

REVIEW OF CASES.

The cases of this disease, under the author's care during the years 1836-37, were too few to furnish by an *analysis* any results worth being recorded.

*Danger arising from the complication with Bronchitis.*—Allusion may however be made to one of the cases, the history of which seemed to shew that the structural alterations resulting from pneumonia in the chronic form and confined to one lung may persist without materially interfering with the general health and compatibly with the active avocations of life, as long as it is not complicated with bronchitis. In the case alluded to, of a robust man æt. 40, a college servant and of rather intemperate habits, there were present, both during the treatment and at the period of his discharge, the usual physical signs, denoting more or less solidification of the lung, viz. dulness on percussion, feeble and distant respiratory murmur, bronchophonia and bronchial respiration in the affected side, and puerile respiration on the sound side—the sputa being at one time glairy and occasionally sanguinolent, and the occurrence of fine crepitation proved that this solidification was the product of pneumonia—but the supervention of the symptoms of bronchitis, at the period of his admission into the hospital, indicated the complication of a bronchial with a parenchymatous affection, and the suffering was then extreme from orthopnœa, &c. This complication yielded to the remedies employed, principally to the antimonial treatment, and the patient returned to his previous state, the physical signs of solidification of the lung still persisting. This condition has remained stationary up to the present time (12 months), the individual continues in his situation and his aspect does not betray the existence of any malady.

*Fatal Termination of the Disease in a Child apparently from gaseous Distention of the Stomach and Bowels.*—One other case deserves notice from the circumstance attending its fatal termination. A child aged 20 months, had been subject to shortness of breath from the time of being weaned (12 months), but was stated to have had cough but three weeks. The child died suddenly five days after becoming an out-patient of the hospital. On its admission the symptoms indicated impeded pulmonary circulation, attended with wheezing cough and occasionally sanguinolent expectoration. For some days previous to its death the child had had a ravenous appetite, which it was allowed to indulge without much discrimination in the choice of food; during the last three or four days the belly had become much distended, and on the day of its death, after a hearty meal, was enormously tympanitic, producing much increase of dyspnœa: in which state the child died rather suddenly. On examination the following day both lungs were found to be solidified posteriorly, portions of them sinking in water and presenting in their section a dark fleshy appearance; the bronchial tubes contained a good deal of muco-purulent secretion without the mucous membrane appearing much congested. The stomach and whole intestinal canal were found enormously distended with gas, the former containing partially digested food in small quantity; the mucous membrane, as far as there was an opportunity of examining it, was apparently healthy.

*Remark.*—It seems probable that, in this case, the distention of the abdomen being suddenly augmented by an improper meal, so impeded the motion of the diaphragm as combined with the solidified state of the lungs to produce sudden death. May not the *ravenousness* of the appetite have been produced by the increased surface of the stomach, consequent on its gaseous distention, preventing the *ingesta* being sufficiently embraced by that viscus, and thus by their presence not being followed by that sensation of *satisfaction,* which results from the due repletion of the stomach? It is well known that this ravenousness is a very common symptom accompanying dyspeptic and hysterical flatulence.

### Pleuritis.

*General Statement.*—This disease occurred still more infrequently than the last in the general practice of the hospital—more than one-half the cases were admitted during the Spring months.

*Review of Cases.*—The cases likewise of this disease, under the author's care, were too few to render their formal analysis profitable—but one or two observations, suggested by a review of them, are subjoined.

### SYMPTOMS.

*Bronchophonia.*—The physical signs of the early stage of pleurisy before any considerable effusion has taken place, it is well known, may be very obscure or inconsiderable. In one case, in which the disease, subjected to treatment very early, was sufficiently acute, and its nature unequivocally denoted by the presence of the pleuritic stitch with high inflammatory symptoms, the only appreciable physical sign that was detected, was bronchophonia over the affected side, while the respiratory murmur continued not sensibly abnormal—the bronchophonia subsided with the general symptoms, and the recovery was complete.

*Creaking Sound accompanying Respiration.*—This sound, resembling that of dry leather, though present in other affections of the chest, seems to be more particularly the accompaniment of that stage of pleurisy in which absorption of the effused products may be supposed to be considerably advanced—it may, as appeared from the cases to which these observations refer, be the only sound heard accompanying the respiratory movement, and may take place of the proper respiratory murmur : coming on in the convalescent stage of the disease, it may remain permanently after complete recovery—it was noted in one instance several months after the discharge of the patient, at a time when there remained no other indication of his having been the subject of any thoracic affection. This sound had none of the "rubbing," "to and fro" character, described by some—it has not occurred to the author to hear the latter description of sound except in pericarditis.

### TREATMENT.

The cases were treated on the ordinary antiphlogistic plan, and presented nothing unusual under this head. One instance may be quoted, in which the employment of calomel and digitalis seemed to contribute mainly to the absorption of the pleuritic effusion, when the late period at which the treatment commenced precluded the use of very vigorous depletion by blood-letting.

### APPENDIX.

The following case is subjoined, as presenting a form of complaint, which the author is not aware has been before observed, or at least narrated, as a sequela of pleurisy.

Case. *Neuralgic Pain and Palpitation succeeding an attack of Acute Pleurisy,* in a robust subject, a coach-porter, æt. 25.

At the time of his admission into the hospital the acute stage of the pleurisy had long subsided, the attack having commenced in the usual way with pain in side, dyspnœa, and fever, fifteen weeks previously, and been treated by the usual remedies. The physical signs, in reference to the left side of the chest, indicated that a pleuritic product of some kind still remained ; there being dulness on percussion, with total absence of the proper respiratory murmur at the infero-posterior aspect of the left lung, where only a "creaking" was heard—in front the respiration was normal on both sides—the dyspnœa was slight, and there was an absence of fever. His principal ailment was severe pain in the cardiac region, extending to the left arm, and reaching down to the fingers, of a lancinating nature, accompanied by palpitation of the heart, and increased by motion of the trunk and deep inspiration. Nothing could be discovered abnormal in the physical phenomena of the heart's action beyond the palpitation. Leeching with perfect rest produced some abatement of the pain, and the palpitation was controlled from time to time by the fresh application of a belladonna plaster, digitalis being given in small doses during the same period. Still further improvement continued on keeping open a blister to the side for a week, but the symptoms again became more severe on his attempting to work in the garden. The pain, which was now described as "dragging," commencing at the cardiac region, took a different direction, extending to the crista ilii—it became again less severe on cupping over the region of the heart—the palpitation, in the mean time, had greatly subsided. As hitherto, the symptoms still indicated the persistance of pleuritic effusion; the patient was put upon a mercurial plan to the extent of producing a smart salivation; after which the respiratory sound was somewhat more developed, but percussion continued dull—he expressed himself however as feeling greatly relieved in his chest—subsequently there was a slight return of palpitation, and he still felt a constant though not severe pain in the cardiac region, extending backwards. A seton was then inserted in the cardiac region, and he was sent into the country, medicine being discontinued. The discharge was maintained for a month; in the mean time his strength was completely restored, but he still felt pain on walking, and had occasional palpitation—auscultation and percussion gave the same results as before—he was soon after enabled to resume his occupation as coach-porter, which he has since continued, and is apparently free from complaint. The treatment just detailed extended from the beginning of February to the middle of May.

The cases of other affections of the pulmonary organs were too few in number, and generally too ordinary in the phenomena they presented, to render either a synoptical review of them, or a selection of instances from among them, serviceable to the purposes of the present communication.

One exception may be made in favor of a case of croup, occurring about the age of puberty, which was memorable both for the extreme severity of the symptoms, and the success attending the vigorous and persevering employment of active remedies.

### LARYNGITIS.

*Case.* G. M. æt. 14, a bricklayer's labourer, was admitted in-patient Jan. 11. 1836, in a state of extreme suffering, presenting all the symptoms of croup in the most aggravated form. The cheeks and lips were of a purple hue, and the general surface very chilly. The respiration exceedingly laborious, with long pauses between each inspiration, and accompanied with the croupy sound—and every now and then there was a loud ringing cough. There was tenderness on pressure over the larynx, and inability to swallow or articulate. The pulse was very irregular; a full and firm beat every now and then, being followed by several smaller and feebler pulsations. He was immediately put into the warm-bath, and 12 leeches applied in the course of the trachea whilst he was in the bath; and the bleeding was afterwards maintained for three hours. The bath restored the temperature of the surface, and increased for a time the force of the pulse, but the croupy symptoms remained the same. Six grains of calomel had been laid on his tongue previous to his admission—mercurial inunction in both axillæ was now carried on vigorously by two assistants. At the end of three hours the improvement was but slight, the temperature being maintained, but the pulse again becoming feeble, although not so irregular—the respirations still continued rare, and the paroxysms of suffocative dyspnœa as violent. Hot water was then applied externally over the larynx and trachea in a sponge half squeezed, and as hot as the nurse could bear, for eight or ten minutes. The inunction was repeated, and a purgative glyster administered, which acted well. By the evening a considerable improvement had taken place; there having been a reaction on the surface and in the pulse—the breathing had become much more tranquil; the paroxysms fewer and much less violent, and he was able to swallow; a grain of calomel was ordered to be given hourly. The remainder of the case need not be given in detail, as the improvement subsequently was uninterrupted and rapid; so much so that in two days from his admission there no longer remained any of the symptoms peculiar to croup. The mercurial treatment was continued during the first 24 hours, and then followed by small doses of tartarized antimony; the gums were only slightly affected; the pulse did not entirely recover its rhythm till a day after the subsidence of the croup. He was reported convalescent on the 16th.

### DISEASES OF THE HEART.

*General Statement.*—Inflammatory and organic affections of the heart in the years 1836-37 furnished no more than 0.6 per cent.

### Rheumatic Affections of the Heart.

*General Statement.*—The participation of the heart in rheumatic affections of the system generally, is by no means of unfrequent occurrence among the patients of this institution, but at present there are no data to establish the proportion of cases presenting this complication.

#### ANALYSIS OF CASES.

*Rheumatic Origin—Form of the Disease.*—As in all the cases the patients were subject to attacks of ordinary rheumatism in various degrees, antecedently to, or contemporaneously with, the affections of the heart, it may be inferred that the latter was in each instance of a rheumatic origin and character. It would appear also from Case 2, (vide Appendix) that *pericarditis* does not uniformly constitute the primary or chief *form* of the cardiac disease in each attack; for in this instance dissection showed that, while the traces of disease of the pericardium indicated only a remote and partial attack of pericarditis, the disease of the mitral valves and interior of the left auricle, indicated an attack of endocarditis, which from the nature of the appearances presented must have been of a much more recent date. Case 4 seems to the author to present a very common form of rheumatic affection of the heart, but which from the milder character of the symptoms has not met with the same consideration as the more acute form.

#### SYMPTOMS.

*"To and fro" Sound.*—In case 1, which from the completeness of the symptoms may be regarded as presenting a perfect type of pericarditis, and has been accordingly subjoined in greater detail, the value of this sound, the "to and fro" sound, was well displayed: its quality, expressive of friction between the two surfaces; the coincidence of its first occurrence with the period of marked subsidence of pain and dyspnœa; its continuance for a short time, a few days only; combine to show that its presence characterizes a stage, a favourable passage in the history and course of the disease—and this instance is quoted in confirmation of the explanation ably given of this sign by Dr. Watson—that it supervenes when the absorption of the effused fluid has advanced so far as to permit the contact of the free and the reflected pericardium, and is the first step towards the adhesion of the two surfaces, an ultimate condition, which may be often considered a fortunate result, as accomplishing the, for the most part, only attainable degree of reparation of which the disease, when extensive, is susceptible.

*Bellows-murmur.*—The subjoined cases intimate, it is conceived, that the occurrence of this phenomenon is by no means invariable, but that every other ordinary symptom may exist to denote the presence of the affection, that the disease may recur several times, and its usual consequences in embarrassing the circulation be observed for a considerable period of time, and yet the sounds of the heart betray nothing abnormal in their quality. This remark appears to be borne out by the cases 4 and 5, in which organic affection of the heart of a rheumatic origin, it can scarcely be doubted, existed. Cases of chronic affections of the heart, may be very severe in respect to other symptoms, yet (as far as the instances under review can establish the fact) be compatible with a return to the ordinary exertions of active life, if they have never been accompanied in their course with a bellows-murmur; and as such a compatibility almost supposes the functional integrity of the valvular structure of the heart, this would appear to favor the notion that all modifications of the sounds of the heart, even a low degree of bellows-murmur, essentially depend on the condition of its valves; and that the force and abruptness of the ventricular contraction can only so far produce this change, inasmuch as such inordinate contraction may virtually interfere with the due action of the valves, for if force and abruptness of contraction were *alone* adequate to the production of such a sound, they would have caused it in the cases 5 and 6, in which they existed as prominent symptoms. (The occurrence of the bellows-murmur in anæmia and chlorosis is by no means repugnant to this view—in which affections, though unattended with textural degenerations, the physical relations subsisting between the muscular and valvular apparatus of the heart and the quantity of blood to be moved, may readily be conceived to be very different from those which obtain in the healthy state of the general system.) With regard to the situation of the bellows-murmur it may be observed that, in one instance (case 3), the intensity of this sound was much greater behind than before; it is possible that this might be accounted for by the condensation of the lung (consequent on the pleurisy which was present as a complication) furnishing a more solid and therefore better conductor of sound.

COMPLICATIONS.

*Pleurisy.*—Inflammation of the pleura seems to have co-existed with the rheumatic affection of the heart in two out of the five cases. In case 3 its presence was unquestionable; in case 1 the shifting of the principal pain at one time to a distance from the præcordial region,* and the occurrence of bronchophony seemed to denote also a pleuritic complication. May it not still be a fact, notwithstanding that some pathologists are at issue with Laennec on this point, that in all cases where the pain is extreme, as in these two instances, the pericarditis is associated with acute inflammation of the neighbouring pleura; and thus, the occasional occurrence of acute pericarditis without pain being a prominent symptom, admit of a ready explanation?

*Pneumonia.*—In addition to the two cases cited as being associated with pleurisy, in a third case (2) the disease was complicated with a double pneumonia, which was the immediate cause of the fatal result.

---

* This circumstance, not stated in the report, occurs in the original notes of the case.

MORBID ANATOMY.

*Endo-carditis.*—The appearances described in case 2, of the mitral valves and internal membrane of the left auricle, it is imagined, must have been the result of recent inflammation; this is inferred from the softness of the deposit, the facility of its removal and its accurate resemblance, especially that of the internal surface of the left auricle, to the recent products of inflammation in other surfaces. The different state of the coagula found in the left, the diseased cavities of the heart, from those occurring in the right, is perhaps not altogether undeserving of notice. It is likewise observable that in this case there was considerable contraction of the auriculo-ventricular opening, although the deposit was soft and apparently recent.

TREATMENT.

*Setons.*—Allusion is made to the treatment adopted in the cases under review, chiefly for the purpose of attesting strongly the value of one remedy, the seton, for although not unfrequently used in conjunction with other means, its importance as a remedy of the first rank in *acute* cases, has not been duly appreciated, and its *early* adoption, has not, it is thought, been sufficiently attended to.

As a remedy in the advanced stages and in the chronic form of the disease, a seton in the præcordial region has obtained credit with most writers, but has not been so readily acknowledged to be serviceable in the very earliest state and most acute form of the disease; yet in four out of the five subjoined cases it was employed with apparently good effect in all; but the most prompt and decided relief ensued from its adoption in the acmé of the most acute of the cases (1). The report but imperfectly conveys the entire change in the condition of the patient, the restoration to comparative comfort, which took place from the moment the discharge from the seton was established; and from that period there was no return of any pain or dyspnœa: it is not implied that the other concurrent remedies were not at the same time both essential and serviceable. A similar instance occurs to the recollection of the author of decided relief to the symptoms in a case in which the state of the patient was still more alarming; it happened many years ago in the practice of Dr. Roberts, at Bartholomew's, in the instance alluded to, with symptoms plainly denoting an affection of the heart, there was combined a typhoid condition, indicated by a black tongue, delirium, &c., but the whole aspect of the case was changed by a fortunate recurrence to a seton, and the patient rescued from imminent danger.

*Iodine.*—This remedy, in the form of the hydriodate of potash, seems, on the showing of the subjoined cases, to merit being ranked high among those of subordinate or secondary importance. It can, still less than the seton, supersede the use of blood-letting and mercury; but in aid of the latter, or when it is desirable to suspend or discontinue its use, iodine seems by no means an insignificant resource. In case 5 it appears to have arrested a third, though slight relapse of the disease.

APPENDIX OF CASES.

Case I. *Acute Pericarditis. Occurrence of the "to and fro" Sound—its Subsidence, Permanence of the Bellows-Murmur: Great Relief from a Seton.*

S. H. aged 18, was admitted in-patient April 4, 1836; she had previously had rheumatic attacks apparently unaccompanied by any affection of the heart, but she was habitually rather subject to palpitation. The present attack commenced a fortnight before her admission, and besides the then existing symptoms, there had been during this time, till within a day or two, slight articular rheumatism.

On her admission the lips were pallid, cheeks injected, and the ground of the complexion sallow—the tongue was moist, covered with a brownish fur in the centre, and red at the tip—the appetite was tolerable and the bowels regular.

She was then suffering severely from pain seated principally in the cardiac region but extending outwardly, increased on inspiration, coughing and twisting the trunk—there was great tenderness on pressing between the 5th and 6th ribs, near their cartilages—the breathing was hurried, the alæ nasi dilating widely on inspiration—the pulse was 128, vibrating, and rather full but soft. The heart beat tumultuously, and a loud bellows-murmur was perceptible under left mamma, synchronously with the first sound.

She had been bled in the arm and leeched and blistered in the side previously to her admission. Calomel and opium were now ordered, at first in large doses (Cal. gr. viij. Op. gr. j.) afterwards it was continued in smaller and more frequent doses, and latterly only to the extent of Calom. gr. j. Opii. gr. ss. three times daily—with this was combined the use of sinapisms applied from time to time to the cardiac region. This plan was persisted in for a fortnight, no ptyalism ensuing. In the mean time the pain and the dyspnœa had on the whole considerably abated, though subject to occasional aggravations which were relieved by the sinapisms—the tenderness was almost entirely removed—the bellows-murmur persisted with some abatement. The pulse was reduced in frequency, generally less jerking, but very excitable—there had all along been cough with some serous expectoration—occasionally slight rheumatic pain and swelling of the joints had occurred; and there had been profuse sweating. Her aspect however had disimproved; her complexion becoming very waxy.

At this stage of her complaint a seton was inserted under the left breast, and from the time that it began to discharge (in about three days), she never had any return of pain, and all urgent dyspnœa had ceased. Three or four days subsequent to the insertion of the seton she was put upon an iodine-treatment in conjunction with the calomel and opium in reduced doses. This plan was continued for 18 days (from the 22d April to the 10th May)—with the above remedies for the greater part of the time was combined the use of digitalis in small doses and mercurial inunction. During this period, new symptoms had declared themselves, and further changes ensued; two days after the seton had begun to discharge, and when the patient's sufferings had greatly and rapidly abated, another sound besides the bellows-murmur was for the first time perceived—this was the "rubbing" the "to and fro" sound—it extended over a large surface, as high as the cartilages of the third and fourth ribs, and across the sternum—it was loudest a little to the right of the left nipple. At this time the chest emitted a dull sound on percussion, in the cardiac region from below the cartilage of the fourth rib, and extending laterally to the outer margin of mamma (about four inches square)—there was slight bronchophony at the roots of both lungs.

In about eight or ten days from the time the rubbing sound was first perceived, it had greatly diminished, and in twelve days was no longer perceptible—the bellows-murmur continuing. In the mean time the seton still discharged, and slight ptyalism had been produced—the general improvement in her condition had been maintained and even progressed.

Towards the conclusion of the period during which this plan, as stated, had been pursued, nausea and vomiting occurred; the iodine, digitalis and mercurials were in consequence discontinued—the inunction was at a subsequent period resumed in reduced doses. The gastric disorder soon ceased—the gums continued sore for about a week, but she was allowed to improve her diet; in about ten days from leaving off the medicine she was able to walk in the hospital garden; and on the 30th of May, eight weeks from her admission she was discharged, but told to attend as an out-patient. At this time the seton still discharged; the bellows-murmur was as loud as ever accompanying the first sound, the second sound of the heart was clangorous, respiration was audible in front of the heart on deep inspiration; she only suffered from slight dyspnœa and palpitation on ascending stairs. No treatment of importance, with the exception of maintaining the discharge from the seton, which was however gradually ceasing, was adopted while she continued an out-patient, and she was finally discharged on the 16th of May, considering herself quite well.

She presented herself at the hospital again in September 1837. In the interval she had married, and had a child—during her pregnancy she felt very well; since her confinement had had rather more palpitation, and was more short-breathed; the bellows-murmur continued, but upon the whole she seemed to be in the enjoyment of good health.

Case II. *Endo-carditis—Double Pneumonia.* R. B. æt. 27, carpenter, spare, and of intemperate habits, was admitted in-patient January 4, 1838. He had two previous rheumatic attacks, the last occurred two years before, from which time he had been short-breathed, and suffered palpitation on slight exertion. The present attack commenced a month previous to his admission, affecting the shoulders, loins and lower extremities, and during the whole month the palpitation had been constant and more violent. At the time of admission the face was pale and the features drawn and the tongue tremulous; the chief pain was down the right side of the trunk and in the right shoulder, and the perspiration was profuse. The impulse of the heart was excessive in the cardiac region, but there was no unusual extent of dulness on percussion; there was a loud bellows-murmur, and the pulse was 90 and small.

Sinapisms were ordered to be applied twice daily over the cardiac region, and Calom. gr. j. Opii. gr. ss. to be taken three times a day.

This treatment, varied in degree, was continued for twelve days, no ptyalism had been produced, and the pain had ceased and the palpitation much abated; but he complained of feeling weak and low, and the remedies were suspended. Shortly after he was attacked with cough, and in two days his condition had greatly deteriorated; the cough was dry and paroxysmal, causing a feeling of constriction at the sternum—no râle was perceived, but the respiratory sound seemed rather in excess in the left lung and in the lower lobe of right lung, while in its upper lobe the murmur was feeble—there was no dulness on percussion.

The impulse of the heart was increased, and the pulse 120 and undulating; the countenance was anxious and the eyelids puffy; leeches were ordered to the præcordial region and a sinapism to the epigastrium. On the following day the symptoms were less distressing, but he was pale and very weak. An occasional small dose of carbonate of ammonia was given to revive him. On the next day, the 20th of January, the dyspnœa and cough were much increased; there was expectoration streaked with blood, and fine crepitation was now heard in the infra-posterior region of the left lung—the heart was beating tumultuously and the bellows-sound very loud; the anxiety of the countenance extreme. In this state he could not be prevented from returning to his own home, where he died the same evening.

*Post Mortem Examination Fourteen Hours after Death.*

A few ounces of straw-coloured fluid were found in the pericardium. A smooth white patch of adventitious membrane covered the pericardium at the anterior surface of the heart, and there were several small tough cellular bands connecting the parts at the base of the heart. The heart was double its usual size, the enlargement being produced principally by the increased dimensions of the left side—the right cavities were, however, dilated; there was but little thickening of the muscular parietes of the left ventricle, while its cavity was much dilated, and was filled as well as the left auricle with soft and uniformly dark coagula—the right cavities contained less coagulum, which was firmer, and some portions free from colouring particles. The mitral valves were studded with soft caruncular excrescences, readily detached from the surface, and extending to and incrusting several of the chordæ tendineæ: a similar deposit, but more distinctly granular, covered the internal surface of the auricle—the left auriculo-ventricular opening would admit only two fingers.

The upper lobes of both lungs, in their central portions especially, were red and indurated (second stage of pneumonia), the lower lobe of left-lung was red and gorged (first stage of pneumonia).

Case III.—*Acute Pericarditis complicated with Pleurisy.*

J. W., aged, very slender and tall, laborer, was admitted in-patient, January 4, 1838. For several years he had been incapable of hard work from being subject to attacks of pain in the chest, and rheumatic affections of the limbs of no great severity. During the whole of the Winter he had been ailing from pain in the left side of the chest, unaccompanied with any cough. A week previous to his admission, his wrists and knees had been swelled and painful—he was extremely emaciated, with a wan and pallid countenance—was breathing quick and laboriously, with dilatation of the alæ nasi, and there was an expression of the greatest anxiety.

The respiratory murmur was puerile in right lung, and more so in upper lobe of the left lung, while opposite to the inferior angle of the left scapula, there was œgophony and bronchial respiration; below this level, on the same side, there was in the postero-lateral aspects of the lung, complete dulness on percussion and absence of respiratory sound. With this there was extreme tenderness on pressure between the sixth and seventh left ribs in the præcordial region—this region was considerably elevated—a loud and very harsh bellows-murmur, accompanied the first sound of the heart. The pulse was 96, weak, small, and soft. He denied ever having suffered materially from palpitation.

Leeches were applied over the region of the heart, and bled freely in a poultice, for a whole night; and antimonial draughts were ordered to be taken frequently.

On the following day the relief that had taken place was striking; all anxiety of expression, and urgency of dyspnœa and tenderness were gone. The treatment for the ensuing 10 or 12 days was chiefly confined to the antimonial mixture, a blister between the shoulders, and latterly mercurial liniment to the left side of the thorax.

All symptoms of acute inflammation of the pleura had subsided from the time of the application of the leeches, and the œgophony had soon disappeared; but dulness on percussion, and absence of respiration in the lower aspect of left lung, with bronchial respiration in other parts, indicated that there still remained effusion in the left pleural cavity. The pain in the region of the heart occasionally threatening to return, leeches were subsequently repeated, and cal. gr. j., opii, gr. ss. ordered to be taken three times a-day, which was continued for nearly three weeks, the antimony having been left off: a seton likewise was about the same time inserted in the præcordial region—from this time there was no return of pain in the chest, and his general health improved greatly: the bellows-murmur was undiminished, and was now remarked to be louder behind than in front: the second sound of the heart was slightly clangorous, and percussion was still dull for the space of about four inches square in the region of the heart: and the conditions before noticed in the inferior portion of the left-side of the chest still remained; in other respects he recovered so as to admit of his being discharged on the 6th of March. On his presenting himself at the hospital some weeks afterwards, though incapacitated for labour, he made no complaint of any suffering.

The physical conditions, as ascertained by the exploration of the chest, remained the same.

Case IV.—*Frequent Attacks of Cardiac Affection, unattended by any Bellows-murmur in a Rheumatic Female.*

M. J., æt. 25, laundress, having embonpoint, florid cheeks, but general complexion and lips pallid, had, for several years before her last admission in March 1837, been subject to rheumatism of a subacute character, accompanied each time with symptoms of affection of the heart, and in the intervals suffering from the same symptoms in a slighter degree.

The principal symptoms of heart-affection were palpitation and inordinate impulse of the heart; pain, with some tenderness in the præcordial region, and dyspnœa of moderate intensity, but at no time was any bellows-murmur to be detected—the urgency of these symptoms yielded to cupping and setons, maintained for a considerable time, in the region of the heart. Since her first attack she has always been subject to palpitation and dyspnœa on a slight exertion, but has still been capable, except at intervals, of pursuing her occupation as laundress.

On her last admission, for general subacute rheumatism, with a recurrence of pain in the region of the heart, the latter readily subsided on the application of leeches.

Case V.—*Rheumatic Affection of the Heart, unattended by any Anormal sound—benefit from Setons, and apparentlg from Iodine.*

H. S., a girl, æt. 13, was admitted in-patient Dec. 10, 1835. She had been subject to palpitation of the heart, and nine months previously had had a rheumatic attack, affecting the right-side of her chest. Five days before her admission, after an accession of rigors and faintness, she was seized with pain at the chest, and at the time of entering the hospital was suffering from wandering pains in the thorax, back, and limbs, increased by warmth in bed, and attended with sweating, but she complained most of pain under the cartilages of the left hypochondrium, extending to the back, and increased by pressure under the hypochondrium and on inspiration; with this there was violent palpitation, and great excitability of the heart, the pulsations being extremely rapid at one time, as on changing from the horizontal to the upright position, and at another, falling below the usual standard of frequency—but there was no anormal sound of the heart, nor any tenderness on pressing between the intercostal spaces; while the impulse in cardiac region was great. Colchicum, sinapisms, cupping, Belladonna plaister were employed, and the pain was removed without any abatement in the other symptoms: the relief to the pain was likewise uncertain, and only yielded for a time to the repetition of the colchicum—a seton was then inserted in the cardiac region, 19 days after her admission, and at the expiration of three weeks, the palpitation was much abated, and, ceasing to suffer any pain, she was made out-patient. But in a few days she was again seized with excessive palpitation, laborious respiration and pain under the left-breast; cupping, castor-oil, cal. gr. j., night and morning, were ordered, and the calomel was continued for about a fortnight; but little amendment ensuing, a second seton was inserted, and in a week's time was attended with the same relief as the first: during a month from the insertion of the second seton, small alterative doses of calomel or blue pill were continued, but no further improvement took place. Iodine, in a mixture, containing the hydriodate of potash, was then substituted for the mercurials (which had never affected the gums), and continued for three weeks, the discharge from the seton being maintained; the symptoms by this time were much further abated, and the palpitation recurred only on extra exertion, and her general health was much improved likewise. A third time there was a partial recurrence of the same train of symptoms, which were again checked by a temporary return to the use of iodine: and she was soon after discharged, and after the interval of several months she was seen in the apparent enjoyment of health.

### Diseases of the Heart not of a Rheumatic Origin.

The cases of other organic affections of the heart, not of a rheumatic origin, of which the notes have been preserved, being but few, and not presenting much correspondence in their symptoms, no analytical review of them is attempted; but reports of some of these as individually deserving some notice, are subjoined, with such remarks as their histories or necroscopical appearances suggested. Cases 1 and 2, as contributions to morbid anatomy are not, it is conceived wanting in interest; the first, as presenting dilatation of the right cavities, added to, and perhaps resulting from, congenital malformation of the semilunar valves of the pulmonary artery, the left side of the heart remaining healthy—the second, as at least a rare instance of disease, viz., ulceration and division of an otherwise healthy semilunar valve from attrition, with the two remaining valves in a state of extreme ossification, and likewise as presenting the co-existence of two distinct forms of disease in the lungs, associated with pulmonary hæmorrhagy.

The author has permission to mention a case, which occurred during the course of last Summer, in the practice of one of his colleagues; presenting a form of cardiac disease, which he believes to be unique in the records of pathology—at least, he has the authority, if he did not deceive himself, of Sir Astley Cooper, who saw the preparation, for its being such.

Case.—*Cavity lined with Adventitious Membrane in the Septum of the Ventricles, and communicating by two Apertures, one recent, one of an older date, with the two Ventricles respectively.*

An old gentleman, the subject of calculus, and in whose bladder many large stones were discovered, died suddenly; and on examining the heart, it was found that, while its texture generally was soft and flabby, there was an appearance of ecchymosis in front, opposite to, and towards the base of the septum of the ventricles: on further examination it appeared that there was in the interior of the septum a small flattened cavity, lined with a granular membrane, and having an aperture about the size of a pea, its edges being even and covered with the same granular sort of membrane, leading into one ventricle; and another small ragged, ecchymosed, and seemingly recent opening into the other ventricle. The heart was otherwise normal.

Short notices of three other cases, although incomplete in their histories, as wanting necroscopical confirmation are added, in consequence, chiefly of the uniformity of the physical signs, which each case respectively offered while under continued observation. While in works written expressly to elucidate the diagnosis of different diseases of the heart, certain assemblages of phenomena are assigned, with more or less precision, for each known form of disease respectively; the occurrence of other associations of physical signs differing from those which have been thus appropriated, may serviceably be recorded as denoting either that there are other forms or complications of organic disease of the heart, of which the collective signs have hitherto not been assigned, or that the representations already given, are in some particulars incomplete and admit of correction. Cases 3 and 4, were of this description, which are offered as presenting rather unusual *combinations* of phenomena, which yet were sufficiently uniform and constant to warrant the belief that they represented some definite and stationary form of organic disease of the heart.

APPENDIX OF CASES.

CASE I.—*Dilatation of right Cavities of the Heart, Patescency of right Auriculo-ventricular opening—Semilunar Valves of Pulmonary Artery, two only*—Tuberculated Lungs—Mucous Membrane of larger Intestines "Mammelonée"—Opacity of Arachnoid.*

R. C. æt. 24, labourer, was admitted in-patient October 6, 1835, of a frame generally robust, but with a mal-formed chest, the sternum projecting, and being flattened laterally. For two years he had been affected with dyspnœa, and sixteen months previously had been discharged from the hospital on recovering from an attack of purulent ophthalmia, but having at that time a cough and being subject to head-aches. On his second admission his complexion was dusky, the eyelids were puffy, and there was a tendency to œdema of the ancles after slight exertion. He had cough and dyspnœa, and expectorated globular, opaque, grey sputa. The pulse averaged 100.

The sound of the heart was very loud in the proper cardiac region, but was loudest under left clavicle—the impulse against the chest was most direct, and strongest between the 4th and 5th ribs, but here it was not excessive. The first sound of the heart was briefer than natural, the second remarkably loud and clangorous, but did not extend far to the right side of the sternum—these conditions of the heart's action continued unchanged to within a few days of his death. The auscultatory signs of the pulmonary disease are not detailed as being irrelevant to the principal object of the report, but it may be stated that there was an entire absence of respiratory murmur in the left side of the chest, which was also universally dull on percussion.

From the time of his admission, and even previously, there was a disposition to drowsiness. At first the pulmonary symptoms attracted the principal attention, for which a variety of remedies were employed without producing any manifest change. On the 4th of November he was attacked with diarrhœa, which, with occasional interruptions, continued the most prominent symptom to the day of his death, which did not occur till the 1st of January, 1837. With the diarrhœa, there was combined considerable tenderness in the right hypochondrium—the usual remedies were employed to combat these new symptoms, but which were productive only of occasional and temporary relief—the legs latterly had become anasarcous, and shortly before his death the heart beat "joggedly," and the dyspnœa increased; the intellect was unimpaired to the last.

*Post-mortem Examination Forty-eight Hours after Death.*

*Head.*—The arachnoid presented on the surface of the hemispheres opaque patches of a glistening white, especially in the vicinity of the longitudinal sinus—glandulæ Pacchioni excessively developed—lateral ventricles somewhat distended.

*Chest.*—The lungs were universally adherent to the parietes of the chest; the pericardium occupied a much larger space in the chest than is natural, and contained about a pint of yellow serous fluid. The external surface of right auricle was rough, with firm coagulable lymph in small quantity. Both cavities on the right side of the heart, but especially the ventricle, were much enlarged, the muscular parietes retaining their natural thickness: the right auriculo-ventricular opening readily admitted four fingers—the tricuspid valves presented no material alteration of structure. The semilunar valves of the pulmonary artery were but two in number and very large; the diameter of the pulmonary artery was considerably greater than that of the aorta. The left side of the heart and the aorta were perfectly healthy.

---

* The heart is preserved in the Museum.

The left lung was in a remarkable degree smaller than the right, and excessively indurated throughout, in some places almost of cartilaginous consistence. It contained tubercles of various sizes, but all immature and of a yellowish grey colour; in their intervals the lung was firmly indurated, and of a reddish-grey appearance. The upper lobe of right lung was in the same condition, the middle and lower lobes were comparatively healthy, containing only a few scattered tubercles. The bronchial membrane was purple and congested.

*Abdomen.* The structure of the liver was degenerated, its vascular network being much atrophied, and its general appearance pale. Throughout the whole tract of the colon the mucous membrane presented a singularly puffy and corrugated appearance (" mammelonnée"), and was firmer than usual: one or two fringed ulcers were found near the rectum—the sigmoid flexure was dragged into the pelvis.

*Remarks.*—As in the above case, both the congenital malformation and the subsequent alteration of structure were confined to the right side of the heart; the record of the physical phenomena attending the action of the heart, which were noted during life, is the more interesting from the rarity of the occurrence itself of the opportunities of similar observation.

The puffiness and dusky hue of the countenance denoted from the first an obstruction to the venous circulation in the chest. The malformation of the chest, and consequently altered relations of the organs contained within it, may account perhaps for the impact of the heart against the chest having been observed to be between the fourth and fifth ribs. The only remaining signs, then, that seem directly referrible to the condition of the right side of the heart, were, the shortness of the first sound, and the ringing quality and loudness of the second sound. The enlargement being exclusively confined to the right side, it may be supposed that the predominance of this side caused *its* sounds to predominate, and to produce the peculiarity observed in these physical signs. The unusual shortness of the first sound may perhaps be accounted for by the dilatation of the right cavities and the valvular patescence, which would render the contraction of the ventricle incomplete, and thus the duration of the passage of the blood into the artery being abridged, the sound accompanying it would be brief likewise. The sounds due to the left side may be supposed to have been obscured—further, as the characters of the second sound were the same in quality which this sound presents naturally, and only exaggerated in degree, this is consistent with the mechanical condition of the semilunar valves of the pulmonary artery, which, natural in texture, were unusually large to make up for their deficiency in number. (Of course this explanation can only apply on the assumption that the action of these semilunar valves during the *diastole* are mainly concerned in the production of the second sound of the heart.)

From the patient's history, in which there was no account of any habitual embarrassment in the circulation in earlier life, and as he was of a robust frame, it seems that the malformation of the valves did not initiate any grave disorder, but was compatible with the due performance of the vital functions, till a new and constitutional disease was set up; the tuberculous diathesis, which by impeding the pulmonary circulation caused an extraordinary demand upon the right side of the heart;—in such a conjuncture the originally imperfect construction of the valves, inadequate to the now obstructed circulation, would obviously cause dilatation of the cavities, and thus conspire with the tubercular disease to hasten the fatal result, which occurred while the tubercles were still crude.

The nature and extent of the lesion observed in the intestinal mucous membrane was connected undoubtedly with the long-continued diarrhœa—and was perhaps a condition analogous to the cellular infiltration of other parts, and a result equally of the embarrassed circulation.

Case II. *Extreme Ossification of two Aortic Valves; Ulceration and Division of the remaining Valve. Pulmonary Apoplexy and Splenization of the Lungs.*

J. B., æt. 36, very robust, and addicted to intemperance, was admited in-patient Jan. 25th, 1838. Nine or ten years previously he had expectorated blood for the first time immediately after a fall, and continued occasionally to spit small quantities in the morning for several years; but all hæmoptysis had ceased for the last two years. Three or four months previous to his admission he had suffered from cough, attended with frothy expectoration; and, when admitted, the most prominent symptoms being those of bronchitis, he was for the first two days treated for that affection. At this period he was seized with a severe fit of dyspnœa and acute pain in the left side of the chest, which was speedily followed by an expectoration of frothy blood in considerable quantity, which continued till within a day or two of his death.

At this time the sounds of the heart were very obscure, the impulse not excessive, the pulse 120, small and intermittent; a vein being opened in the arm, the blood flowed with extreme difficulty from a large orifice. On the 30th, for the first time, a slight bellows-murmur was thought to be perceptible in the cardiac region. The difficulty of breathing amounting to orthopnœa, with great restlessness and agitation, became more and more distressing—the lower extremities rapidly and largely infiltrated, bullæ forming under the cuticle. On the 1st of February slight cerebral disturbance supervened, and he had the aspect and manner of a person half-intoxicated; the features were altered and shrunken, and his strength rapidly failing, he died on the following day, (February 2d.)

The details of the auscultation of the lungs are omitted for brevity's sake; they agreed generally with the post-mortem appearances; percussion could not be adequately performed in consequence of there being great tenderness of the chest. The detail of the treatment, which was totally unavailing, is likewise omitted; it consisted mainly in blood-letting and the use of tartarized antimony, and subsequently of acetate of lead.

Post-mortem examination, 22 hours after death.

*Exterior.*—Lower extremities anasarcous; posterior aspect of body mottled with purple maculæ.

*Abdomen.*—The liver, without being much enlarged, was much altered in its form: the upper surface of the right lobe being elevated into a somewhat conical shape, and thrusting the diaphragm high into the chest. This had produced dulness on percussion over a considerable space in the right side of the chest. Its section presented the "nutmeg" appearance, and its vessels were much congested. The remaining abdominal viscera were healthy.

*Thorax.*—About a pint of bloody serum was contained in the two pleuræ. The *heart,* preserved in the museum, was much enlarged, being more than double its natural size—its point rounded; there were also some small white patches on its pericardial surface, one of which was flocculent but tough. All the cavities, but especially the ventricles, were enlarged to about twice their natural dimensions, their parietes retaining their ordinary thickness and being tolerably firm. No valvular disease existed on the right side. The *mitral* valves were thickened, but not ossified, retaining their pliancy. Two of the *semilunar* valves presented an extraordinary amount of ossification, being blended into one scabrous mass, which seemed to the finger directed from the ventricle to the aortic opening like a rough fragment of mortar; the opening appeared almost occluded, the point of the finger being scarcely admissible. On laying open the arch of the aorta, it was found that the third semilunar valve was not ossified, but had been divided by ulceration down its center into two pendulous flaps, the division being opposite the roughest and most projecting portion of the ossified mass. The aortic arch presented atheromatous patches; the whole of the internal surface of all the cavities of the heart and of the arch presented an uniform redness.

*The Lungs.*—The mucous membrane of both lungs was throughout congested, but of a darker hue in the left; the bronchi and their ramifications contained a quantity of frothy blood. *Right Lung.*—Upper and middle lobes crepitant, and the posterior aspect of all the lobes moderately congested, but a portion of about two inches square of the thin anterior edge presented externally a dark purple appearance, and its section was black and resembled a sliced coagulum of blood. *Left Lung.*—The upper lobe crepitant and comparatively healthy; the inferior lobe in its lateral and posterior aspect was entirely solid and friable; the section of the greater portion of this lobe resembled very accurately the spleen in colour and somewhat in texture, while an abundant quantity of purple, prune-colored fluid was very readily expressed from it; the remaining smaller portion, accurately and abruptly defined from the former, presented the same apoplectic appearance as that described in the right lung, and pure blood oozed from its surface on gentle pressure.

*Remarks.* In reviewing the history of the above case in reference to the necroscopical appearances, it appears possible to trace what was latterly the probable sequence of organic changes which eventuated in the entire destruction of the mechanism of the circulation. The amount of ossification of the aortic valves was so great that the disorganizing process must have been for a long time in activity. The postponement of the inevitable result of a fatal impediment to the circulation was probably owing to the circumstance of one of the three semilunar valves having been exempt from ossific degeneration; so that although the channel must have long been greatly contracted for the passage of the blood, yet there still subsisted the conditions for securing the onward movement of what little was transmitted, and thus its fatal remora in the lungs was continually prevented; but the ulceration of the remaining unossified valve consequent on the chafing of the opposed bony spiculæ, terminating in its division into two pendulous flaps, must, it is evident, have given the "coup de grâce" to the heart as an organ capable at once of receiving a quantity of blood "a tergo" and transmitting the same onwards, in short all valvular property was then lost, and the disorganization of the heart thus consummated, quickly induced the impediment to the circulation through the lungs, causing extravasation into their cellular and interstitial structure and terminating in death.

It was remarkable that, with so large an amount of valvular disease, the physical signs declaratory of its presence should have been so faint and obscure, and especially that it should remain doubtful to the last whether there was any bellows-murmur or not; but this apparent anomaly admits of ready solution, as the extreme smallness of the aperture must have counteracted the effect of its roughness in producing any rasping sound, by admitting the passage of but a very small quantity of blood at a time.

With respect to the morbid appearances in the lungs, a question arises whether the two kinds of solidification described are to be regarded as two distinct kinds of lesion, or whether they be different stages of the same disease, *i. e.* whether the purple solidification, splenization, from which the prune-colored exudation took place, was an advanced stage of the primary apoplectic state, and that in it the extravasated blood had undergone some ulterior change; or whether it was pneumonia in its passage into the third stage of Laennec. The author is not aware of any case in which these two appearances are described as having co-existed; although mention is made by the translator of Laennec, in a note to the Chapter on pulmonary apoplexy, of the splenization of the lung, by whom it is regarded as the result of the chronic form of pulmonary apoplexy.

Case III.—*Hypertrophy of the Heart—supposed Disease of the Aortic Valves—Extreme Venous Discolouration of the Surface—rasping sound—the interval between the Impulse at the Chest, and the Rasping observed to have increased with the Duration and probable Advance of the Disease.*

In W. C., a robust waterman, æt. 28, the following symptoms were observed with but little variation during upwards of three months.

*General Symptoms.*—Distention and dragging at the epigastrium, which caused his principal suffering; dyspnœa on exertion; occasional œdema; nocturnal attacks resembling incubus, followed by pain in occiput.

*The Circulation.*—Extreme venous discolouration of the surface, especially of the face, hands, and feet, without apparent distention of the larger veins or pulsation of the jugulars. The right radial pulse much smaller and more feeble than the left: a corresponding difference thought to exist between the right and left carotids, temporals, &c. respectively. The pulse generally feeble but not irregular.

Impulse of the heart uniformly inordinate, raising the head of the observer applied to the chest; frequent palpitation.

Dulness on percussion over an increased space, about two inches square, in præcordial region, but respiration audible in front of the heart on a deep inspiration.

A rasping heard in cardiac region, over the sternum, carotids, and in the back, varying in extent at different times, yet always loudest just to the left of the sternum in the third intercostal space, and in the latter situation apparently not distant from the surface; synchronous, or very nearly so, with the impulse at the chest, and followed by a second sound, which was very clear, sharp, and occasionally clangorous.

This patient seen again at the interval of 10 or 12 months, was much in the same state generally; the complexion retaining its venous hue, had become more sallow, and cough was added to the other symptoms.

The physical signs referrible to the circulation were essentially the same, but the impulse of the heart had apparently increased, and the rasping sound now very perceptibly *followed* the impulse at the chest, between which and the radial pulse there was also a very appreciable interval.

*Remark.*—Although the rasping was, latterly at least, not synchronous with the impulse at the chest, it was nevertheless the accompaniment or modification of the *first* sound of the heart, as it was followed by the second, which retained its naturally brief character, but was more resonant and ringing. The diagnosis conjectured was—1st, disease and contraction of the aortic valves principally. This opinion was founded on the rasping appertaining to the first sound, and where loudest, its being comparatively near the ear, and on the pulse being weak and small, and at the same time not irregular (vide Hope on Diseases of the Heart). It differed, however, from some of the particulars assigned by Dr. H. as characteristic of this form of disease, viz. the sound was not loudest opposite the middle, but at the left margin of the sternum, it was also not synchronous with the impulse at the chest. 2dly. Dilatation with hypertrophy of the heart, as denoted by the increased space, over which there was dulness on percussion, and the increased impulse. And 3dly, the want of correspondence in the force of the arterial pulse on the right and left side of the head and upper extremities might depend upon an extension of the disease to the arch of the aorta, in which the arteria innominata might be implicated at its origin.

The circumstance of the interval between the impulse at the chest and the rasping of the first sound having *increased sensibly,* during the 12 months intervening between the times of observation, constituted the chief interest of the case. It may perhaps be thus accounted for—the aortic obstruction had increased during this period, while the propulsive force of the hypertrophied ventricle had not been exalted in the same proportion, and thus the passage of the blood through the opening was longer in being accomplished; hence the interval between the *commencement* of the ventricular systole, as denoted by the impulse at the chest and its *completion* coincident with the cessation of the rasping had become prolonged.

The embarrassment of the respiration, it may be observed, at no time corresponded in *degree* with the extent and intensity of the venous discoloration, if that depended upon any impediment to the pulmonary circulation; it was more analogous to the appearance presented where there is a communication between the arterial and venous sides of the heart.

CASE IV. *Very loud and superficial Bellows-murmur behind the Sternum not synchronous with the Arterial Pulse.—Obscurity of Sounds and Feebleness of Impulse in the proper Præcordial Region.*

L. B. a travelling saleswoman, æt. 30, who had been for some time a surgical patient for ulceration of the scalp, resulting from an injury, was transferred to the care of the physician in February last. The symptoms most urgent at that time had reference to an enlargement of the spleen, accompanied with intestinal disorder, and shortly afterwards she had a severe attack of acute pleurisy in the left side. It was on convalescence from this last affection that the attention was principally directed to symptoms indicating disease of the heart or large vessels.

She had been subject to palpitation for two or three months, but of no great severity; neither, independently of the pleuritic attack, did it appear that at any time there had been any very great embarrassment to the pulmonary circulation. There was no venous hue of the complexion, which was for the most part pale, especially the lips.

The pulse was large, tense, and regular.

Percussion of the chest in præcordial region dull over a considerably increased space. Systolic impulse very feeble, being with difficulty perceptible. Impulse above the clavicles, but unaccompanied with any thrill. Both sounds of the heart in præcordial region audible but not distinct. A loud and prolonged bellows-murmur heard in præcordial region, but loudest opposite the middle of the sternum, where it seems very superficial; heard also above the clavicles, especially above the right; the murmur was not synchronous with the pulse, whether felt above the clavicle or at the wrist, but seemed to take the place of the second sound. So feeble was the impulse at the chest, that its synchronism or anachronism with the morbid sound could not be satisfactorily determined. The above symptoms remained stationary while she remained under observation, a period of several weeks.

*Remark.*—The association of physical signs just enumerated, which were constant, do not agree with the assemblage assigned by Dr. Hope to any of the principal forms of valvular disease, either of the aortic or mitral valves.

The situation of the bellows-murmur where loudest, its nearness, and the regularity of the pulse, agree best, according to Dr. H.'s diagnosis, with disease of aortic valves or ascending aorta; but the coincidence of the murmur with the second sound, or rather its want of synchronism with the arterial pulse, do not accord with the presence of such a form of disease.

Again, the apparent coincidence of the murmur with the second sound of the heart agrees with the diagnosis offered by Dr. H. of disease of the mitral valves, but its nearness to the ear, the situation where it was heard to be loudest, and the regularity of the pulse, and, it may be added, the comparatively slight embarrassment to the pulmonary circulation, militate strongly against such a diagnosis in the present case.

